# Hémangiome fusiforme: une localisation inhabituelle

**DOI:** 10.11604/pamj.2016.24.90.8479

**Published:** 2016-05-27

**Authors:** Fatima Zahra Nasreddine, Bouchra Baghad, Soumiya Chiheb

**Affiliations:** 1 Service de Dermatologie CHU Ibn Rochd, Casablanca, Maroc

**Keywords:** Hémangiome fusiforme, tumeur bénigne, excision chirurgicale, Spindle-shaped hemangioma, Benign tumour, Surgical excision

## Abstract

L'hémangiome à cellules fusiformes été décrit par Weiss et Enzinger en 1986 et anciennement nommé hémangioendothéliome à cellules fusiformes. Depuis l'avènement des études immunohistochimique il n'est plus considéré en tant qu'angiosarcome de bas grade. C'est une tumeur bénigne vasculaire. Il touche presque exclusivement le derme des extrémités distales. Nous rapportons le premier cas avec localisation au niveau de l'omoplate, du sein, des cuisses et de la mandibule. Selon la littérature, seulement 9 cas localisés au niveau de la tête et le cou ont été rapportés. Nous rapportons un nouveau cas de cette entité rare et mal connue qui peut prêter confusion avec les tumeurs malignes. Notre patient avait une localisation au niveau de l'omoplate, du sein, des cuisses et de la mandibule. Une biopsie-exérèse était réalisée. L’évolution a été favorable avec un recul de 6 mois, sans rechute.

## Abstract (en)

Spindle cell hemangioma, formerly known as spindle cell hemangioendothelioma, was described by Weiss and Enzinger in 1986. Since the advent of immunohistochemical studies it is no longer considered as low grade angiosarcoma. It is a benign vascular tumor It almost exclusively affects the dermis at the distal ends. We report the first case of a patient with spindle cell hemangioma located in the scapular, breast, thighs and mandibular area. According to the literature, only 9 cases located in the head and neck were reported. We report a new case of this rare and poorly understood entity that can be confused with malignant tumors. Our patient suffered from spindle cell hemangioma located in the scapular, breast, thighs and mandibular area. He underwent excisional biopsy. The evolution was favorable with 6-month follow up, without relapse.

## Introduction

L'hémangiome à cellules fusiformes a été décrit par Weiss et Enzinger en 1986 et anciennement nommé hémangioendothéliome à cellules fusiformes [1]. Depuis l'avènement des études immunohistochimiques, l'hémangiome à cellules fusiformes n'est plus considéré en tant qu'angiosarcome de bas grade [2]. C'est une tumeur bénigne vasculaire qui survient à tout âge chez les deux sexes sous forme de nodule rouge brunâtre dermique ou sous cutané unique ou multiple au niveau des extrémités [3]. Des localisations extracutanées ont été rapportées dans la littérature: les viscères, la moelle épinière et la cavité buccale. Elle correspond à une lésion vasculaire faite de larges vaisseaux à lumières labyinthiques d'aspect caverneux. L'endothélium est aplati et les thrombi peuvent être retrouvés dans ces espaces caverneux. Cependant, une étude récente a mis en évidence une coloration positive focale de Prox1 dans l´hémangiome à cellules fusiformes d'où la probabilité d'une origine lymphatique [4]. L'exérèse chirurgicale doit être complète pour éviter les récidives. Nous rapportons un nouveau cas de cette entité rare et mal connue qui peut prêter à confusion avec des tumeurs malignes

## Patient et observation

**Figure 1:**
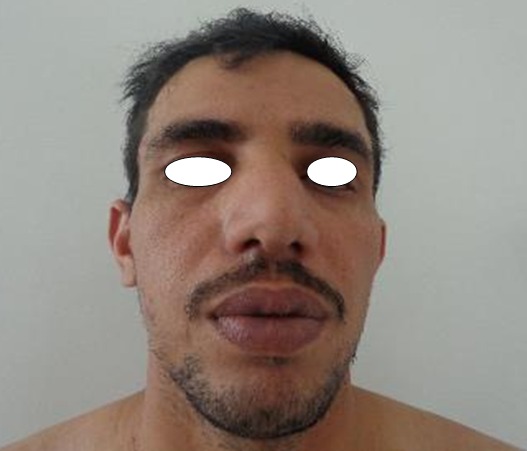
Nodule sous cutané de la mandibule

**Figure 2:**
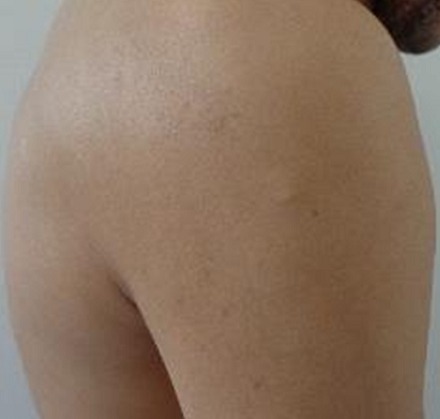
Nodules sous cutané du bras gauche

**Figure 3:**
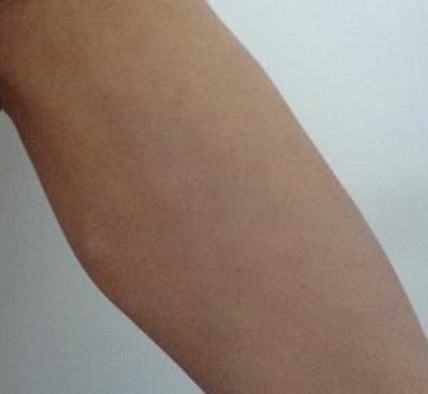
Nodule sous cutané du bras gauche

**Figure 4:**
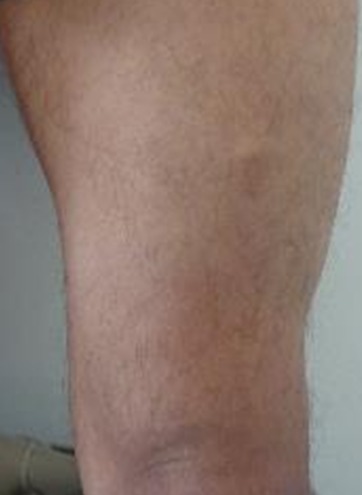
Nodule sous cutané de la cuisse gauche

**Figure 5:**
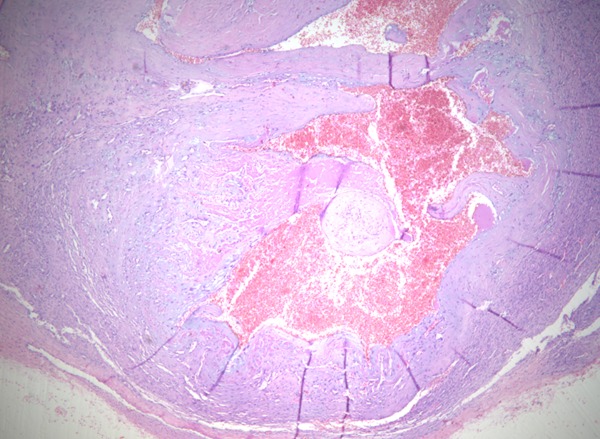
Faible grossissement thrombose remanié

**Figure 6:**
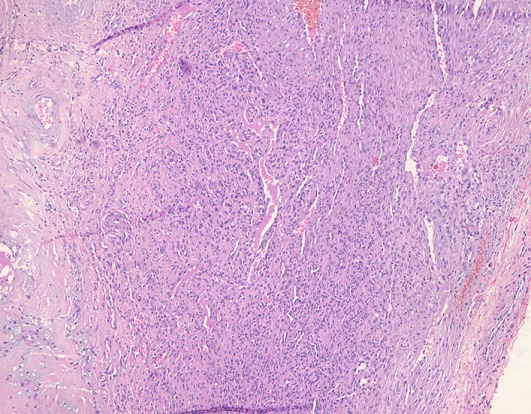
Aspect histologique

Un homme de 34 ans sans antécédents pathologiques particuliers présentait depuis 8 ans des nodules sous-cutanés douloureux au niveau de l'omoplate gauche avec apparition progressive de nouveaux nodules au reste du corps. L'examen clinique retrouvait des nodules sous-cutanés de consistance élastique avec une peau en regard normale, de tailles variables de 1,5cm en regard de la mandibule (Figure 1), de (4cm /2cm) en regard de l'omoplate gauche, deux nodules de 1cm au niveau du bras gauche (Figure 2, Figure 3) et des 2 cuisses (Figure 4). Aucune adénopathie n’était palpable. Les diagnostics de léiomyome, léiomyosarcome, métastases cutanées, sarcoidose, de tuberculose cutanée, de syphilis tertiaire et de kaposi ont été évoqués. Un bilan sanguin comprenant une numération formule sanguine a montré une hyperleucocytose à prédominance PNN (PNN=7830). L'IDR à la tuberculine était positive à 25mm. Les BK crachats étaient négatifs. Le reste du bilan notamment le dosage de l'enzyme de conversion de l'angiotensine, la sérologie syphilitique et la sérologie VIH étaient normaux. La radio thorax était normale. L’échographie des parties molles a montré des lésions hypoéchogènes d’échostructure identique de contours arrondis homogènes et vascularisés. La biopsie exérèse des lésions nodulaires a objectivé des lésions assez bien limitées pseudo-encapsulées; correspondant à des lésions vasculaires faites de larges vaisseaux à lumières labyinthiques d'aspect caverneux. L'endothélium est aplati, régulier sans atypies, sous tendu par une paroi fibreuse hyalinisée, certaines lumières comportent des thrombi fibrino-cruoriques remaniés (Figure 5). En périphérie de la lésion, on retrouve des vaisseaux capillaires plus petits adossés les uns aux autres donnant un aspect fuso-cellulaire (Figure 6). L’étude immunohistochimique a objectivé l'absence d'expression de l'HHV8 par les vaisseaux et les cellules fusiformes. Celles-ci expriment le CD34 évoquant un hémangiome caverneux avec composante fuso-cellulaires sans signes de malignité. Le patient a bénéficié d'une exérèse complète des nodules sous-cutanés.

## Discussion

L’ hémangiome à cellules fusiformes est une tumeur bénigne rare qui peut se voir chez le nouveau né ou l'enfant. Cependant, l'apparition est décrite chez l'adulte d’âge moyen avec une prédilection pour les extrémités [3]. Notre patient avait une localisation au niveau de l'omoplate, du sein, des cuisses et de la mandibule. Selon la littérature, seulement 9 cas localisés au niveau de la tête et le cou ont été rapportés [5, 6]. Scott et a présenté un cas d'HCF avec des nodules situés au niveau de l'oreille, des doigts, du pénis et de l'avant bras chez un homme de 70 ans [7]. Baron et al a rapporté un HCF localisé au niveau du bord latéral gauche du nez d'un nourisson de 17 mois [5]. Ces lésions vasculaires se présentent le plus souvent sous forme de nodules dermiques ou sous-cutanés indolores uniques ou multiples prenant le plus souvent le même segment de membre [8]. Elles sont souvent diagnostiquées avec retard vue l'absence de signes objectifs locaux. Notre patient a consulté après 8 ans. Les formes bilatérales ou disséminées sont rares [9]. Approximativement 10% des cas sont associés à d´autres anomalies ou syndromes, y compris les varices, le lymphoedème, le syndrome de Klippel-Trenaunay-Weber et le syndrome de Maffucci [3]. Chez notre patient aucune association n'a été retrouvée. Histologiquement, l'HCF est constitué de nodules composés de zones cavitaires constituées de vaisseaux à paroi fine séparées par des cloisons fibreuses. Les espaces endothéliaux caverneux, en nid d'abeille. Les cloisons peuvent être plus épaisses et contenir des cellules fusiformes. Il n'y a pas d'atypies nucléaires. Les cavités vasculaires dilatées peuvent contenir des thrombi ou des phlébolithes [9]. Les avancées en immunohistochimie ont permis une identification de l´origine des cellules fusiformes notamment une expression du CD 31, du CD 34 et du factor VIII. Le D2-40, le Glut-1 et HHV-8 ne sont pas exprimés [9]. Le diagnostic différentiel de l'HCF se porte essentiellement avec le sarcome de Kaposi et avec d´autres types d´hémangiomes incluant le caverneux, l'histiocytoide, les hémangiomes capillaires lobulaires disséminés et l'hémangiome congénital non involutif qui peut présenter des nodules faisant protrusion dans les lumières vasculaires, l´angiomatose épithélioïde, l´hémangioendothéliome kaposiforme et l´hyperplasie endothéliale papillaire intravasculaire [7]. La combinaison des espaces endothélio caverneux avec les thrombi organisés, la multiplication de cellules fusiformes, l´activité mitotique minimale et la négativité de l'HHV8 permettent d’éliminer les autres diagnostics et ceci à l’étape histologique [2]. Malgré que l'hémangiome à cellules fusiformes soit considéré comme une lésion bénigne [8], il peut récidiver sous forme de lésions multiples après excision, tout en défigurant l'extrémité entière [9]. Le traitement repose sur l'excision chirurgicale large des lésions [7]. Les excisions limitées récidivent certainement, mais il n´y a aucun consensus sur l´incidence des récidives [7] et il est difficile de distinguer une récidive d'une progression de lésions inapparentes de petite taille laissées en place. Notre patient a bénéficié d'une exérèse complète des lésions sans récidive avec un recul de 6 mois. La radiothérapie, l´interféron à faible dose a-2b et intralésionnel et l´administration intraartérielle d´interleukine-2l recombinante et la rapamycine a eu du succès dans le traitement des lésions diffuses et les lésions qui sont difficiles à exciser chirurgicalement [10]. Il n´y a eu aucune mortalité rapportée dans la littérature. Un cas de métastase ganglionnaire régionale après 40 ans a été rapporté [3, 6]. La régression spontanée est possible. À la lumière de ces données, il est important de prévenir le patient ayant un hémangiome à cellules fusiformes du risque élevé des récidives et fournir le suivi approprié.

## Conclusion

L'hémangiome à cellules fusiformes est une tumeur cutanée bénigne et rare qui siège essentiellement au niveau des extrémités. L'atteinte diffuse est exceptionnelle. Il faut rechercher systématiquement les associations avec le syndrome de Maffuci. Le traitement repose sur l'excision large pour éviter les récidives avec une surveillance régulière des patients.
